# Mutational frequency of *KRAS, NRAS, IDH2, PIK3CA*, and *EGFR* in North Indian gallbladder cancer patients

**DOI:** 10.3332/ecancer.2017.757

**Published:** 2017-08-07

**Authors:** Aarti Sharma, Ashok Kumar, Niraj Kumari, Narendra Krishnani, Neeraj Rastogi

**Affiliations:** 1 Department of Surgical Gastroenterology, Sanjay Gandhi Post Graduate Institute of Medical Sciences (SGPGIMS), Lucknow, Uttar Pradesh, 226014, India; 2 Department of Pathology, Sanjay Gandhi Post Graduate Institute of Medical Sciences (SGPGIMS), Lucknow, Uttar Pradesh, 226014, India; 3 Department of Radiotherapy, Sanjay Gandhi Post Graduate Institute of Medical Sciences (SGPGIMS), Lucknow, Uttar Pradesh, 226014, India

**Keywords:** gallbladder cancer (GBC), formalin-fixed paraffin-embedded (FFPE), genetic mutations

## Abstract

**Background:**

Gallbladder cancer (GBC) has a peculiar geographical distinction, with a high prevalence seen in North India and Chile. There are various aetiopathogenetic mechanisms of GBC causation; one of them is a series of pathogenic mutations, which is responsible for the malignant transformation of gallbladder epithelium. Therefore, the present study aimed to find out cancer-specific hot spot mutations in five major cancer-related genes *KRAS* exon1 &2, *NRAS* exon1, *IDH2* exon, *PIK3CA* exon 20, *IDH2* exon 4 and *EGFR* exon 20 in North Indian GBC patients and their association with clinicopathological variables.

**Material and methods:**

This study included 34 histopathologically confirmed GBC cases. The clinical material consisted of formalin-fixed paraffin-embedded (FFPE) blocks of the patients. DNA isolation was done from FFPE tissue. DNA sequencing was performed by the capillary electrophoresis method. The chi-square (χ^2^) test was used to test for a statistically significant relationship between two categorical study variables.

**Results:**

The overall incidence of somatic mutations in *KRAS* exon 1&2, *NRAS* exon1, *IDH2* exon4, *PIK3CA* exon20, and *EGFR* exon 20 in Indian GBC patients was found in 8/34 (23.5%), 3/34 (8.8%), 4/34 (11.7%), 7/34 (20.6%), 7/34 (20.6%), respectively. *KRAS* exon 1 and two mutations were found to be significantly associated with advanced stage GBC patients.

**Conclusion:**

*KRAS*, *PIK3CA*, and *EGFR* were found to be the most frequently mutated genes among the five tested in this study.

## Introduction

Gallbladder cancer (GBC) has a peculiar geographical distinction, with a high prevalence observed in North India and Chile [1]. However, its frequency in western countries is relatively less [2]. GBC accounts only for 0.5% of all gastrointestinal malignancies cancers in the United States [3]. The 2000–2013 figures from the Surveillance, Epidemiology, and End Results (SEER 18) show that age-adjusted incidence rate of GBC in 2013 is 1.1528 per 100,000 in the Asian population https://seer.cancer.gov/. Surgical resection remains the only chance of cure, but it is possible in only a small percentage of patients with GBC who are diagnosed early. The overall five-year survival rate for GBC is 32% but for advanced stage, it declines to 10% [4, 5].

There are many pathogenesis means that have been proposed for the occurrence of GBC, which include chronic inflammation, environmental factors, dietary changes, and genetic factors. The less understood aspect for gallbladder pathogenesis is genetic alteration. From the earliest observation that abnormal gene expression could result from a single mutation that can drive a series of events and finally leading to malignant transformation, the mutation continues to evolve as a central mechanism in cancer biology.

The role of various genetic mutations in GBC is a field of active research nowadays. Somatic mutations occurring in intracellular signalling pathways cause aberrant activation of the signalling molecules. These mutations have transformed the diagnosis and treatment in some cancers [6–8]. Some of these genetic changes are associated with particular risk factors, whereas some changes are associated with differences in prognosis [9]. Different subtypes of cancer harbour specific gene mutations that act as invaluable markers for disease diagnosis and prognosis, for example, the leukaemia cells of patients with chronic myeloid leukaemia (CML) contain a mutated gene called BCR-ABL.

The identification of key genetic mutations in GBC that could be involved in carcinogenesis may help to better understand the molecular basis of pathogenesis and possibly help in improving treatment strategies. The mutations of *RAS (KRAS* and *NRAS), IDH2, EGFR,* and* PI3K* are clinically relevant and well associated in many other cancers [10–15]. Therefore, the present study aimed to find out cancer-specific hot spot mutations in five major cancer-related genes *KRAS* exon1 &2, *NRAS* exon 1, *PIK3CA* exon 20, *IDH2* exon 4, and *EGFR* exon 20 in North Indian GBC patients and their association with clinicopathological variables through a hospital-based study.

## Methodology

### Sample collection and DNA isolation

In this study, we have included 34 histopathologically confirmed GBC cases, which were treated in the Department of Surgical Gastroenterology and Radiotherapy. Formalin-fixed paraffin-embedded blocks of the patients, stored as archival material in Department of Pathology were taken as clinical material, without any compromise with histopathological quality. Tumour area was identified by staining a three micron thick section with haematoxylin and eosin, and then, the slides were visualised microscopically. The tumour area having greater than 80% tumour cells was marked. For DNA isolation, we cut some other 20 micron thick sections from the same block using microtome and collected on a glass slide. The slide was fixed on a hot plate at 65° for one to two hours. These sections were then deparaffinised with two washes of xylene followed by rehydration with a graded alcohol (30%, 50%, 70%, 100%) washes. The tumorous area was scarped using a sharp scalpel. DNA extraction was done using commercially available Quigen FFPE DNA extraction kit (Qiagen, Valencia, CA) as per manufacture instructions.

### DNA quality control

DNA concentration was determined using spectrophotometer (Nanodrop^TM^, Thermo Scientific, USA). DNA having minimum concentration of 200 ng/μl was considered for further analysis.

### Primer designing

For primer designing we used primer3 software (http://primer3.sourceforge.net/). The desired sequence of the gene of interest was taken from the NCBI database against which the software gave four sets of primers. The primer pair < 200 bp in length, with annealing temperature between 48–58°, and GC content not exceeding 60% was selected for the polymerase chain reaction (PCR). All primers were binding at a site 70–80 bp away from mutation of interest so interpretation of mutational region was proper and free from noise. The primer sequences used for sequencing analysis are given in Table 1.

### DNA sequencing

PCR amplification products generated by the Lightcycler PCR were purified using QiaQuick reagents (Qiagen, Valencia, CA) and were cycle sequenced using Big Dye v3.1 reagents (Applied Biosystems, Weiterstadt, Germany) according to the manufacturer’s protocol. Sequencing products were purified with CleanSEQ Sequencing Purification System (Agencourt Bioscience Corp., Beverly, MA) and automated sequencing performed by capillary electrophoresis on an ABI3700 (Applied Biosystems). Sequences were aligned and examined by two separate approaches: electronically with a set threshold of 10% and by visual inspection of the electropherogram, using Finch TV software (http://www.geospiza.com/ftvdlinfo.html).

### Statistical and in silico analysis

All the data obtained by the experiments also with clinical details were analysed using SPSS version 16.0 (SPSS, USA). Descriptive statistics of study subjects are either represented as mean with standard deviation or frequency with percentages. The chi-square (χ^2^) test was used to test for a statistically significant relationship between two categorical study variables. A cut-off of 0.05 for *p* values was used as a limit of significance to predict the associations. In addition, the functional consequences of studied mutations were enquired from online webserver COSMIC (http://cancer.sanger.ac.uk/cosmic) that uses FATHMM algorithm (Functional analysis through hidden Markov Models) to predict the mutation impact. Any scores above ≥ 0.7 were classified as ‘Pathogenic’.

## Results

### Characteristic profile of the study subjects

This study included 34 GBC cases which included eight (23.5%) males and 26 (76.5%) females with the mean age of 53.21 ± 10.32 (range 35–71 years). Follow-up data were available for 16 patients only who continued their post-surgery treatment in the Department of Radiotherapy. Follow-up data were collected every three months from the time of enrolment until death or last scheduled follow-up. Characteristics of GBC patients along with all the details regarding treatment response, drug-related toxicity, tumour grade, etc. are listed in Table 2.

### Mutations in KRAS oncogene

*KRAS* (Kirsten ras oncogene homolog) gene belongs to mammalian ras gene family. Any single-nucleotide change can lead to substitution of an amino acid, which finally is responsible for an activating mutation.

We evaluated a total of eight mutations present in exons 1 and exon 2 in *KRAS* oncogene in 34 GBC by performing PCR and DNA sequencing. *KRAS* mutations were determined in 8/34 (23.5%) cases; most of the patients have exon 1 mutations, only two cases have presence of exon 2 mutations (Table 3).

### Mutations in NRAS oncogene

*N-RAS* (neuroblastoma RAS viral oncogene homologue) is a proto-oncogene which encodes a membrane protein that acts as a shuttle between the Golgi apparatus and the plasma membrane. Mutations in this gene have been seen to be associated with rectal cancer, follicular thyroid cancer, etc. [11, 12].

We sequenced the exon 2 of *NRAS* gene to look at the hotspot mutations, that is, p.G12S, p.G12C and p.G12V, which have already been reported as pathogenic in cosmic database. The variants of p.G12V and p.G12C as two hotspot mutations of the *NRAS* exon 1 were found only in 3 out of 34 tumours. No p.G12S, mutation was seen in any of the GBC patients Table 3.

### Mutation in PIK3CA gene

*PIK3CA* (phosphatidylinositol 3-kinase) gene plays a key role in cell growth, survival, proliferation, motility, and morphology. This gene has also been reported as oncogenic and involved in cervical cancer [13].

Here, we have sequenced exon 20 of *PIK3CA* gene. It was found that *PIK3CA* p.H1047R(c.3140A>G) mutation was present in 5/34 GBC patients. Whereas two cases were harbouring p.H1047L(c.3140A>T) mutation (Table 3).

### Mutation in IDH2 gene

Isocitrate dehydrogenase gene codes for a digestive enzyme that catalyses the oxidative decarboxylation of isocitrate to 2-oxoglutarate in the citric acid cycle. This gene is associated with glial tumours [14].

We sequenced exon 4 Of *IDH2* gene to look for the pathogenic mutations that are already reported in cosmic database in GBC patients. Only four cases were positive for p.R140Q c.419G>A mutations. Other pathogenic mutations of exon 4, that is, p.R140W, c.418C>T p.R140L, c.419G>T, p.R140G c.418C>G were absent in all studied GBC cases (Table 3).

### Mutation in EGFR gene

*EGFR* is a cell surface protein that codes for epidermal growth factor receptor protein. It has a major role in cell proliferation. Mutations in this gene are found to be associated with Lung cancer [15].

We characterised one non-synonymous, pathogenic mutation p.T790M c.2369C>T in exon 20 of 7/34 GBC patients (Table 3).

Out of all studied mutations, the most common somatic mutations detected were *KRAS* exon 1 and 2 in 8/34 (23.6%) cases followed by *PIK3CA* exon 20 in 7/34 (20.6%) and EGFR exon 20 in 7/34 (20.6%) cases. *NRAS* exon 1 and *IDH2* exon 4 were found mutated in least number of GBC cases (8.8% and 11.8%, respectively) (all data shown in Table 3).

### Association of gene mutations with clinicopathological data

We examined the relation between gene mutation status and various clinicopathological features, including age at diagnosis, degree of differentiation, stage of tumour and gender. There was no significant association of any of the studied mutations with clinicopathological features except that *KRAS* exon 1 and 2 mutations were found to be significantly associated with advanced stage GBC patients (Table 4).

## Discussion

Occurrence of mutations can be responsible for the malignant transformation of gallbladder epithelium. The tumour causing genes can belong to either tumour suppressor or oncogenes or even DNA repair genes. Any error in any one of the genes can become mutations, which may eventually lead to cancer. GBC also involves the changes in multiple oncogenes and tumour suppressor genes. In future, many of these mutated genes can also represent targets for novel therapeutic agents that are more specific, more efficacious, and less toxic than broad-based chemotherapeutic regimens.

In the present series, we have studied the frequency of pathogenic mutations in five major cancer-related genes in north Indian GBC patients. Population-based data show that North India has high prevalence of GBC [1]. To study the mutational frequency of most common cancer-related genes, we performed Sanger sequencing of 34 GBC patients. The overall incidence of somatic mutations in *KRAS* exon 1 and 2, *NRAS* exon 1, *IDH2* exon 4, *PIK3CA* exon 20, and *EGFR* exon 20 in Indian GBC patients was found to be 8/34 (23.5%), 3/34 (8.8%), 4/34 (11.7%), 7/34 (20.6%), 7/34 (20.6%), respectively. Though most of the mutations were not linked to stage or grade of the disease,* KRAS* exon 1 and 2 mutations were found mainly in cases with stage 3 and 4.

A genetic model for gallbladder carcinogenesis by S. G Barreto *et al* has also shown the presence of *KRAS* mutations as an event of GBC progression to advanced stage [16]. However, many other genetic alterations observed not only in advanced cancers but also in pre-neoplastic lesions depends on type and severity of the tumour [17]. The presence of loss of heterozygosity and *TP53* mutations are the most frequent and early events in GBC (~50%) and they are observed even in normal-like tissues exposed to gallstones [18]. Epigenetic silencing of *CDKN2A (p16)* has been observed in early pre-neoplastic lesions [19, 20]. Based on the above literature, it is clear that the genetic features of GBC change with respect to the stage of cancer and this difference may explain the poor prognosis in advanced cases.

Any mutation in the* KRAS* oncogene that causes the altered expression of translated protein can cause cancer formation. In the literature, *KRAS* mutations have been reported in leukaemia, colorectal cancer, [10] pancreatic cancer [21], and lung cancer [22]. A previous study by Roa J C *et al* has found *KRAS* codon 12 mutations in 30% of GBC cases, which is consistent with our results [23]. However, Ajiki T *et al.,* reported a higher incidence of KRAS mutations (i.e. 59%) [24]. The published literature shows that the frequency of KRAS mutations in GBC ranges from 8% to 80% which indicates the heterogenic nature of cancer [23–29].

The* PIK3CA* gene has also been found to be oncogenic and is well implicated in many cancers, like cervical cancer [30], breast cancer [31]. In the present study, we have sequenced exon 20 of the *PIK3CA* gene. The incidence of somatic pathogenic mutations present in *PIK3CA* exon 20 was 20% (14.7% have H1047R and 5.8% have H1047L change). A study performed in the Chilean population detected* PIK3CA* exon 20 (both H1047R and H1047L) mutations in 37.4% of GBC cases [32]. A research article published by Deshpande *et al* found *PIK3CA* exon 9 mutations in 18.8% GBC [33]. Another study by Zhao S *et al* reported the lower incidence of 6.15 % of *PIK3CA* exon 9 mutation in GBC patients [34]. However, in the last two articles, authors have not found any *PIK3CA* exon 20 mutations in GBC cases.

Similarly, previous studies have shown that mutations in the *NRAS* gene, which lead to a constantly active *NRAS* protein, are found in approximately ~13–25% of metastatic melanoma patients [35–37]. In the present study, we found* NRAS* exon 1 mutations in 3/34 (8.8%) of GBC cases. A previous study by Deshpande *et al* found *NRAS* exon 1 mutations in 6.3% of GBC cases [33].

Mutations that lead to *EGFR* overexpression (known as upregulation) or overactivity have also been reported previously in various cancers including GBC with variable frequencies like squamous-cell carcinoma of the lung (80% of cases), glioblastoma (50%) and epithelial tumours of the head and neck (80–100%) [38]. *EGFR* overexpression has also been reported in 87.5% GBC patients of North American ethnicity [39]. The somatic mutations involving *EGFR* can lead to its constant activation causing abnormal expression of EGFR protein which ultimately drives the neoplastic conversion of a cell. [15]. Our study has found *EGFR* exon 20 mutation in 7/34 (20.6%) cases.

Mutation in *IDH2* has been frequently found in cancers like gliomas and acute myeloid leukemia. In the present study, we found *IDH2* gene mutation in 4/34 (11.7%) of cases. The literature has not found much support for its association with GBC till now. Milind Javle *et al* has found *IDH 1* gene mutation in 4/57 (7%) of GBC cases [40]. But on the contrary, a study by Darrell R. Borger *et al* found *IDH* gene hotspot mutations in cholangiocarcinoma but not in GBC [41].

The plausible explanation behind the variable results reported by various previous studies may be due to the Inter and intra-tumoural heterogeneity of cancer, which describes the observation of different tumour cells showing distinct morphological and molecular profiles including variable gene expression.

The role of mutations in cancer is well defined in the literature. Mutations are also designated as a hallmark of cancer, which allows a cell to invade and metastasise to other body parts. Multiple subtypes of a histopathologically common cancer make it crucial to understand the commonalities and differences among various types and subtypes. Presently, we had studied the distributions of mutation frequencies in five major cancer-related genes and establish their links to treatment outcome. In light of this, our study illustrates that despite being a histologically common entity of GBC (adenocarcinoma of gallbladder), there is heterogeneity with respect to the presence of mutations. *KRAS, PIK3CA*, and *EGFR* gene mutations were more common in GBC as compared to NRAS and IDH2. Moreover, KRAS mutations were also found to be significantly associated with an advanced stage of GBC.

The present study is based on a mutational analysis of five major cancer-related genes. We used micro-dissected gallbladder tissue with greater than 80% of tumour cells as a study material that imparts a positive strength to our results; still we had the limitation of a small sample size. Future studies with a higher number of samples are needed to better define genetically distinct subsets of cancers.

## Conclusion

The present study found the presence of somatic mutations in *KRAS, NRAS, PIK3CA, IDH2,* and* EGFR* in GBC. None of these mutations were significantly associated with clinicopathological parameter except for *KRAS* exon 1 and 2 mutations with advanced stage of GBC. A more robust and larger study is further required to validate these findings.

## Conflict of interest

The authors declare no conflict of interest.

## Author contribution

Aarti Sharma and Dr Ashok Kumar designed, carried out the study and wrote the manuscript. Dr Niraj Kumari and Dr Narendra Krishnani provided the histopathological specimens and helped in tumour area micro-dissection work. Dr Neeraj Rastogi provided the clinical data.

## Figures and Tables

**Table 1.** Primer sequences for PCR.

Gene/mutation ID	Primer sequences	Amplicon length
*KRAS* exon 1	F-5′-CCAGACTGTGTTTCTCCCTTCT-3′ R-5′-AAAGAAAGCCCTCCCCAGT-3′	151 bp
*KRAS* exon 2	F-5′ CATGTTCTAATATAGTCACA 3′ R-5′ AACAAGATTTACCTCTATTG 3′	175 bp
*NRAS* exon 1	F-5′-CTCGCCAATTAACCCTGATT-3′ R-5′-TGGTGGGATCATATTCATCTACA-3′	157 bp
*IDH2* exon 4	F-5′-TCTGTCCTCACAGAGTTCAAGC-3′ R-5′-CTAGGCGTGGGATGTTTTTG-3′	120 bp
*PIK3CA* exon 20	F-5′-AACTGAGCAAGAGGCTTTGG-3′ R-5′-TGTGGAATCCAGAGTGAGCTT-3′	158 bp
*EGFR* exon 20	F-5′-CTCCAGGAAGCCTACGTGAT-3′ R-5′-GTCTTTGTGTTCCCGGACAT-3′	124 bp

F: forward primer; R: reverse primer.

**Table 2.** Characteristics of GBC patients.

Total GBC patients *n* = 34	
**AGE (years)**	
Mean	53.21
Median	54.50
Mode	62
St. Deviation	53 ± 10.32
Range	35–71
**Characteristics**	**No (*n*%)**
**Gender**	
Male	8 (23.5%)
Female	26 (76.5%)
**Gall stone**	
Present	11 (32%)
Absent	23(68%)
**Tumour grade/Degree of differentiation**	
Grade 1 Well differentiated	26 (70.3%)
Grade 2 Moderately differentiated	5(13.5%)
Grade 3 Poorly differentiated	3(8.1%)
**Stage**	
Early (Stage 1)	10(29%)
Late (Stage 2–4)	24(71%)
**Total followed up GBC patients *n* = 16**	
**Gender**	
Male	5(31%)
Female	11(67%)
**Treatment outcome**	
Responders (PR)	12(75%)
Partial and No Responders	4(25%)
**General adverse events and distribution**	
No–mild (0–1)	12(75%)
Moderate–severe (2–4)	4(25%)

GBC: gallbladder cancer.

**Table 3.** Overview of gene mutations found in GBC patients.

Gene name	Nucleotide change	Codon change	FATHMM prediction	Effect		No of patients with mutations
*KRAS *Exon1	c.35 G>T	p.G12Q	0.98	Missense/Pathogenic		5
	c.35G>A,	p.G12D	0.98	Missense/Pathogenic		1
	c.34G>T	p.G12C	0.98	Missense/Pathogenic		Nil
	c.38G>A	p.G13D	0.98	Missense/Pathogenic		Nil
	c.37G>T	p.G13C	0.98	Missense/Pathogenic		Nil
*KRAS* exon2	c.182A>T	p.Q61L	0.98	Missense/Pathogenic		2
	c.182A>G	p.Q61R	0.98	Missense/Pathogenic		Nil
	c.183A>T	p.Q61H	0.98	Missense/Pathogenic		Nil
**Total frequency of KRAS exon 1&2 mutations**						**23.5% (8/34)**
*NRAS* Exon 1	c.34G>A	p.G12S	0.9	Missense/Pathogenic		Nil
	c.34G>T	p.G12C	0.92	Missense/Pathogenic		1
	c.35G>T	p.G12V	0.92	Missense/Pathogenic		2
**Total frequency of NRAS exon 1 mutations**						**8.8% (3/34)**
*PIK3CA* Exon 20	c.3140A>G	p.H1047R	0.96	Missense/Pathogenic		5
	c.3140A>T	p.H1047L	0.96	Missense/Pathogenic		2
	c.3139C>T	p.H1047Y	0.95	Missense/Pathogenic		Nil
	c.3129G>T	p.M1043I	0.97	Missense/Pathogenic		Nil
**Total frequency of PIK3CA exon 20 mutations**						**20.6% (7/34)**
*IDH2* Exon4	c.419G>A	p.R140Q	0.98	Missense/ Pathogenic		4
	c.418C>T	p.R140W	0.90	Missense/Pathogenic		Nil
	c.419G>T	p.R140L	0.99	Missense/Pathogenic		Nil
	c.418C>G	p.R140G	0.92	Missense/Pathogenic		Nil
**Total frequency of IDH 2 exon 4 mutations**						**11.8% (4/34)**
*EGFR* Exon 20	c.2369C>T	p.T790M	0.94	Missense/Pathogenic		**7**
**Total frequency of EGFR exon 20 mutations**						**20.6% (7/34)**
**Total mutations detected**					**29**	

GBC: gallbladder cancer.

**Table 4.** Relationship of detected mutations with clinicopathological features in GBC patients.

Para-meters	Category	Number(%)	*KRAS* exon 1&2			*NRAS* exon 1			*PIK3CA* exon 20			*IDH2* exon			*EGFR* exon 20		
			Yes (%)	No (%)	*p* val	Yes (%)	No (%)	*p* val	yes	no	*p* val	yes (%)	No (%)	*p* val	yes	no	*p* val
Age	> 50	13(38)	25	75	0.3	33	67	0.6	15	85	0.4	50	50	0.4	29	71	.4
	<50	21(62)	42	58		39	61		19	81		37	63		41	59	
Sex	M	8(24)	25	75	0.6	0	100	0.4	36	17	0.2	25	75	0.4	29	71	.5
	F	26(76)	23	77		26	74		64	83		15	85		22	78	
Stage	Early	10(29)	0	100	**0.04**	0	100	0.4	46	22	0.2	10	90	0.4	29	71	.6
	Late	24(71)	39	61		32	68		54	78		21	79		30	70	
Gall stone	No	11(32)	25	75	0.5	33	67	0.2	64	70	0.5	18	82	0.6	71	29	.5
	Yes	23(68)	65	35		71	29		36	30		18	82		67	33	

GBC: gallbladder cancer.

Significant *p* value in bold.
